# Impact of diabetes mellitus and hemoglobin A1c level on outcomes among Chinese patients with acute coronary syndrome

**DOI:** 10.1002/clc.23373

**Published:** 2020-05-19

**Authors:** Ran Xiong, Liu He, Xin Du, Jian‐Zeng Dong, Chang‐Sheng Ma

**Affiliations:** ^1^ Department of Cardiology, Beijing Anzhen Hospital Capital Medical University Beijing China; ^2^ National Clinical Research Center for Cardiovascular Diseases, Beijing Anzhen Hospital Capital Medical University Beijing China

**Keywords:** acute coronary syndrome, diabetes mellitus, hemoglobin A1c

## Abstract

**Background:**

The impact of different glycemic control conditions on in‐hospital and long‐term outcomes among patients with acute coronary syndrome (ACS) is less well defined.

**Hypothesis:**

Diabetes mellitus (DM) with different admission hemoglobin A1c (HbA1c) levels (different glycemic control) could affect outcomes among Chinese patients hospitalized as ACS.

**Methods:**

We categorized 8961 Chinese ACS patients into one of the following three groups: “no DM” (group 1, n = 3773; no DM history and admission HbA1c < 6.5%), “DM with optimal control”(group 2, n = 2241; DM with admission HbA1c < 7.0%), “DM with suboptimal control”(group 3, n = 2947; DM with admission HbA1c ≥ 7.0%). The primary outcome was in‐hospital major adverse cardiovascular events (MACEs). 6098 patients were followed for a median of 3.85 years. Adjusted associations of these three groups with in‐hospital MACEs and long‐term mortality were determined.

**Results:**

DM with suboptimal control (group 3) was associated with greater in‐hospital MACEs (OR 1.46, 95% CI 1.17‐1.81, *P* = .001) than “no DM” (group 1). DM patients (group 2 and group 3) also had higher in‐hospital MACEs (OR 1.42, 95% CI 1.16‐1.73, *P* = .001) than “no DM” patients (group 1). It showed no significantly different in‐hospital MACEs between optimal (group 2) and suboptimal (group 3) control group (OR 1.06, 95% CI 0.84‐1.34, *P* = .63). Both optimal control (group 2) and suboptimal control (group 3) had a higher long‐term mortality (HR 1.26, 95% CI 1.02‐1.56, *P* = .03; HR 1.42, 95% CI 1.16‐1.73, *P* = .001).

**Conclusions:**

ACS patients with DM were associated with higher in‐hospital MACEs and long‐term mortality. Moreover, lower HbA1c level seems to have limited impact on cardiovascular events and long‐term mortality in this high‐risk population.

## 1 INTRODUCTION

In China, more than 700 000 deaths each year, one quarter of all deaths, are caused by coronary events.^1^, ^2^ Rapid epidemiological transition and a concomitant increase in the prevalence of major risk factors have led to an increasing numbers of patients being admitted to hospitals with acute coronary syndrome (ACS). More than two thirds of the burden of death and disability from ACS, which is a major contributor to national mortality and economic burden in our country, will occur in adults aged <65 years.^3^, ^4^

In 2010, an estimated 6.4% of the world's adult population (approximately 285 million individuals) had diabetes mellitus (DM), and the prevalence is projected to increase to 7.7% (approximately 439 million individuals) by 2030.^5^ DM is considered to be a “coronary heart disease (CHD) equivalent” and associated with a 2‐ to 4‐fold increased risk of cardiovascular disease (CVD).

The measurement of hemoglobin A1c (HbA1c) provides a reliable reflection of the glycemic control in the previous 8 to 12 weeks and is minimally affected by stress during ACS. The International Expert Committee has recommended the use of HbA1c in diagnosing diabetes with a cutoff value of 6.5%.^6^ The recommended guideline for patients with CVD is HbA1c values <7%.^6^ Although the benefit of controlling HbA1c levels (glycemic control) in patients with type 2 diabetes on microvascular events such as retinopathy, neuropathy, or nephropathy is well established, the association between glycemic control and macrovascular or cardiovascular events is less well defined.^7^, ^8^ Moreover, most previous studies generally evaluated the impact of diabetes on outcomes in patients after acute myocardial infarction.^9^, ^10^, ^11^, ^12^ Further understanding of the impact of normal and different admission glycemic control conditions on in‐hospital and long‐term outcomes among patients with ACS is essential. In this study, we aimed to investigate the impact of the diabetes and admission HbA1c levels (glycemic control conditions) on in‐hospital major adverse cardiovascular events (MACEs) and long‐term mortality in a large cohort of Chinese patients hospitalized for ACS who underwent modern treatments in the contemporary post‐2000 era.

## 2 METHODS

### 2.1 Study population

The study population was drawn from the China ACS Registry Study, which was both a planned retrospective registry trial and a real world study that sought to investigate the impact of clinical quality and treatment strategy on the short‐term and long‐term outcomes among Chinese patients hospitalized with ACS. The China ACS Registry Study recruited 38 hospitals from three provinces and municipalities (Beijing, Henan, Jilin) throughout North China, including 19 level‐2 hospitals (broadly defined as regional hospitals providing medical services to several communities) and 19 level‐3 hospitals (broadly defined as hospitals providing high level specialist medical services to several geographic regions). The inclusion criteria were local patients admitted with a diagnosis of ACS and aged ≥18 years. Exclusion criteria were having severe non‐cardiac comorbidities with a life expectancy <12 months, transferring from non‐selected hospitals, death in <10 min after hospitalization and non‐local patients. Data of patients' demographic characteristics, history of diseases, symptoms and signs, treatment and in‐hospital events were recorded at baseline.

**FIGURE 1:**
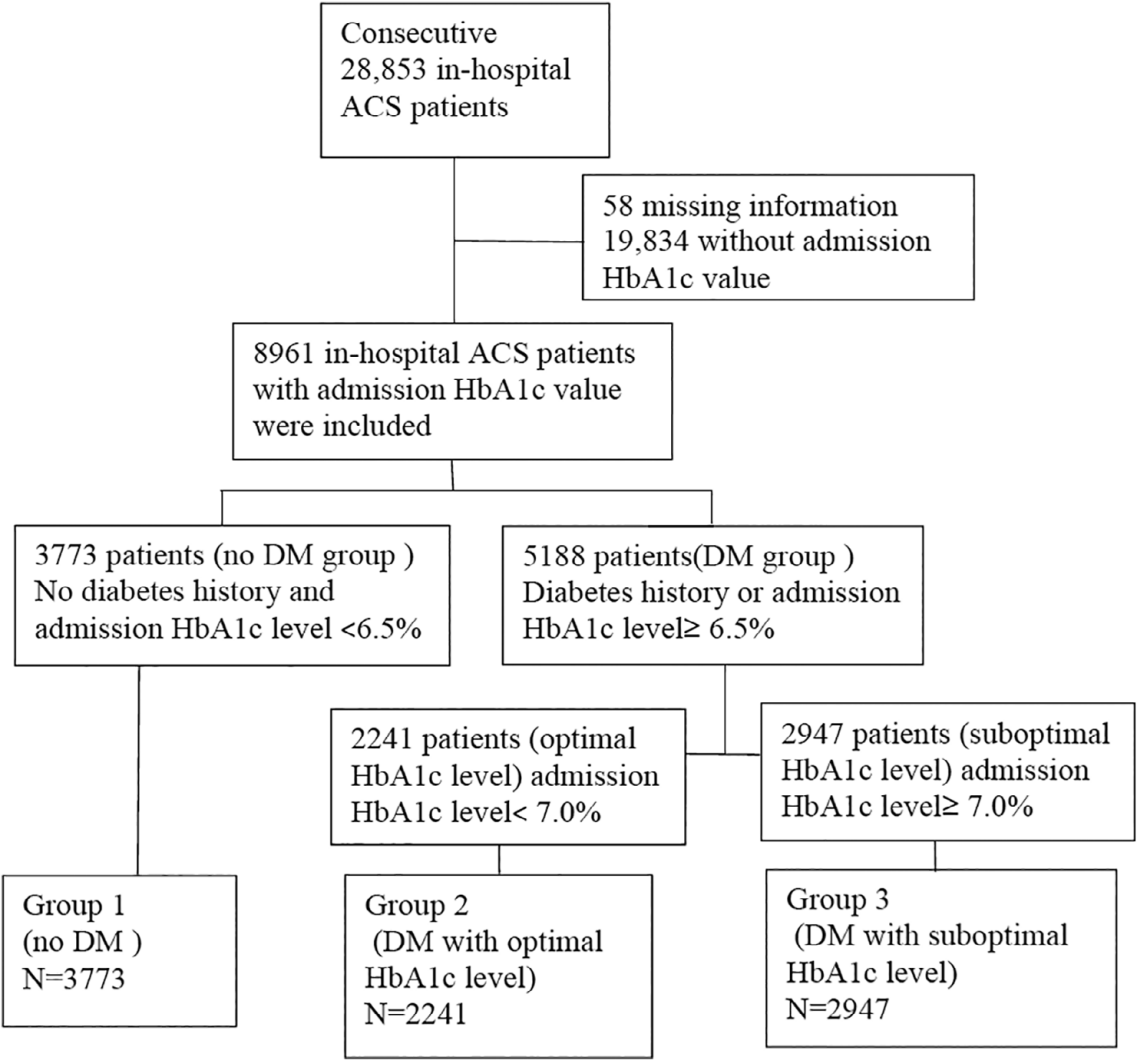
Flow chart of study population

Totally, the China ACS Registry Study recruited 28 853 in‐hospital ACS patients from the year 2008 through 2015, among which 12 640 were from Beijing, 8650 from Henan, 7505 from Jilin and the remaining 58 with missing domicile information. Excluding 19 834 patients without admission HbA1c values, our study comprised 8961 in‐hospital ACS patients with admission HbA1c values. Since guidelines recommended a cutoff value of 6.5% for diagnosing DM and optimal HbA1c values <7.0% for DM patients. Of the 8961 patients, 3773 (42.1%) patients, who had HbA1c levels <6.5% and no history of DM, were classified as no DM (group 1, n = 3773; no DM history and admission HbA1c < 6.5%). The remaining 5188 patients (57.9%), who had definite DM history or admission HbA1c level ≥ 6.5%(newly diagnosed DM), were defined as DM group. Furthermore, 2241 patients in DM group, who had HbA1c levels <7.0% in DM group, were classified as DM with optimal control (group 2, n = 2241; DM with admission HbA1c < 7.0%). The rest of the 2947 patients in DM group, who had HbA1c levels more than or equal to 7.0%, were classified as DM with suboptimal control (group 3, n = 2947; DM with admission HbA1c ≥7.0%; Figure 1).

In particular, 1529 patients who had no previous history of DM, but had elevated HbA1c level ≥ 6.5% (newly diagnosed DM), were included in those 5188 patients as DM group. In these 1529 patients, 873 patients who had HbA1c levels <7.0% were assigned into group 2 (DM with optimal control), the rest 656 patients who had HbA1c levels ≥7.0% were included in group 3.

The following data regarding long‐term all‐cause and cardiac mortality of 6201 patients lived in Beijing were identified through the database of Beijing Centers for Disease Control and Prevention (Beijing CDC). Most of these patients (n = 6098) were followed for a median of 3.85 years (3.85 ± 2.14).

### 2.2 Ethical standards

The medical charts of eligible patients were reviewed to collect requested information by centrally trained and certified research personnel who were not involved with the clinical care of the patients. The study protocol complied with the Declaration of Helsinki and was approved by the Ethics Committee of all hospitals involved.

### 2.3 Clinical outcomes

The primary outcome of this study was the in‐hospital MACEs comprising cardiac mortality, heart failure, non‐fatal reoccurred myocardial infarction, and non‐fatal stroke. Second outcomes were long‐term all‐cause and cardiac mortality: (a) long‐term all‐cause death and (b) long‐term cardiac death.

## 3 STATISTICAL ANALYSIS

Demographics, baseline clinical characteristics, and event rates according to diabetes and HbA1c levels were summarized using means with SD or medians with 25th and 75th percentiles for continuous variables and percentages for categorical variables. Categorical variables were compared using the χ^2^ test, and continuous variables were compared using either one‐way ANOVA or Kruskal–Wallis test, as appropriate.

Multivariable logistic regression models were created to compare in‐hospital MACEs within optimal control (group 2) and suboptimal control (group 3) subgroups, setting no diabetes patients (group 1) as reference, after adjustment for potential confounders associated with in‐hospital MACEs including all baseline and clinical characteristics shown in Table 1, for example, gender, different subtypes of ACS, Global Registry of Acute Coronary Events (GRACE) risk score, history of old myocardial infarction (OMI), history of stroke/transient ischaemic attack (TIA), risk factors of cardiovascular disease and in‐hospital treatments.

**TABLE 1:** Baseline and clinical characteristics stratified by diabetes and HbA1c levels

	Totaln = 8961	No DM (group 1) n = 3773	Optimal control (group 2) n = 2241	Suboptimal control (group 3) n = 2947	*P* value
Age, mean (*SD*), years	63.3 (12.2)	62.1 (12.8)	65 (11.6)	63.4 (11.7)	<.0001
Male gender (%)	65	71.1	60.6	60.5	<.0001
HbA1c, median (IQR)	6.2 (5.7, 7.5)	5.7 (5.4, 6)	6.3 (5.8, 6.6)	8.3 (7.5, 9.5)	<.0001
Diagnose (%)					
STEMI	50.3	55.1	41.8	50.4	<.0001
NSTEACS	49.7	44.9	58.2	49.5	<.0001
Risk factors (%)					
Ever smoker	50.2	55.3	45.3	47.2	<.0001
Hypertension	63.4	55.3	72.7	66.6	<.0001
Diabetes	40.8	0	61	77.7	<.0001
Hyperlipidaemia	21.5	19.7	25	21.1	<.0001
Previous history (%)					
OMI	10.2	9.6	10.7	10.7	.230
PCI	11.1	10.4	12.6	11	.025
CABG	1.3	0.7	1.9	1.6	<.0001
CAD	16.4	14.8	18.6	16.8	.001
Stroke/TIA	14.2	12.1	16.1	15.5	<.0001
eGFR, median (IQR)	110.9 (89, 134.9)	112.3 (92.6, 134.8)	106.8 (84.1, 129.6)	112 (88.1, 139.5)	<.0001
LVEF <40% (%)	4.7	3.4	5.4	6.1	<.0001
Severity at presentation					
Killip class >II (%)	19.9	18.4	18	23.3	<.0001
Heart rate ≥ 100 bpm (%)	7.5	5.9	5.9	10.7	<.0001
SBP < 90 mmHg (%)	1.5	1.8	1.4	1.3	.246
GRACE risk score ≥ 140 (%)	35.6	36.1	33.1	37	.011
In‐hospital treatment (%)					
ASA	96.8	97	95.7	97.4	.002
Clopidogrel	88.5	89.7	87.3	88.1	.013
ACEI/ARB	68	64.8	69.2	71.1	<.0001
Beta‐blocker	77	77.5	75.7	77.4	.230
Statin	96.9	98	95.6	96.5	<.0001
LWMH	79.2	79.9	75.7	80.8	<.0001
Thrombolytic therapy	3.8	4.0	2.9	4.3	.0278
PCI	54.5	59.4	49.1	52.5	<.0001
CABG	1.1	0.9	0.9	1.3	.232

Abbreviations: STEMI, ST elevation myocardial infarction; NSTEACS, non‐ST elevation acute coronary syndrome; DM, diabetes mellitus; OMI, old myocardial infarction; PCI, percutaneous coronary intervention; CABG, coronary artery bypass graft; CAD, coronary artery disease; TIA, transient ischaemic attack; eGFR, estimated glomerular filtration rate; IQR, interquartile range; LVEF, left ventricular ejection fraction; SBP, systolic blood pressure; GRACE, Global Registry of Acute Coronary Events; ASA, acetylsalicylic acid; ACEI/ARB, angiotensin‐converting enzyme inhibitors/angiotensin‐receptor blockers; LWMH, low‐molecular‐weight heparin.

Multivariable Cox proportional hazards models were used to assess the association of diabetic control status with long‐term mortality, using no diabetes patients (group 1) as the reference group. Multivariable adjustment for long‐term mortality considered variables associated with long‐term mortality in the GRACE risk score, and variables from previous modeling in Clinical Pathways for Acute Coronary Syndromes in China (CPACS) risk score, which were considered as confounders and adjusted in previous models.

Associations of diabetic control status with in‐hospital and long‐term outcomes are presented as ORs or HRs with their 95% confidence intervals (95% CIs). A *P* value <.05 was considered significant for all two‐sided tests. All statistical analyses were performed using SAS version 9.4 (SAS Institute, Cary, NC).

## 4 RESULTS

### 4.1 Baseline characteristics

Baseline characteristics stratified by DM and HbA1c levels are shown in Table 1. In general, patients with DM (group 2 and group 3) were older, more were female, had more hypertension, hyperlipidemia, and had more previous coronary artery disease (compared with group 1). Severity of ACS as assessed by presentation with Killip class >II, heart rate ≥ 100 bpm, and GRACE risk score ≥ 140 was markedly more common among patients with DM and suboptimal control (group 3). Although patients in all groups received similar medical and invasive therapy according to contemporary guidelines, patients with DM (group 2 and group 3) tended to be treated with percutaneous coronary intervention (PCI) less frequently, and received angiotensin‐converting inhibitors or angiotensin receptor blocks at admission more often than patients with no diabetes (group 1).

### 4.2 In‐hospital MACE

**FIGURE 2:**
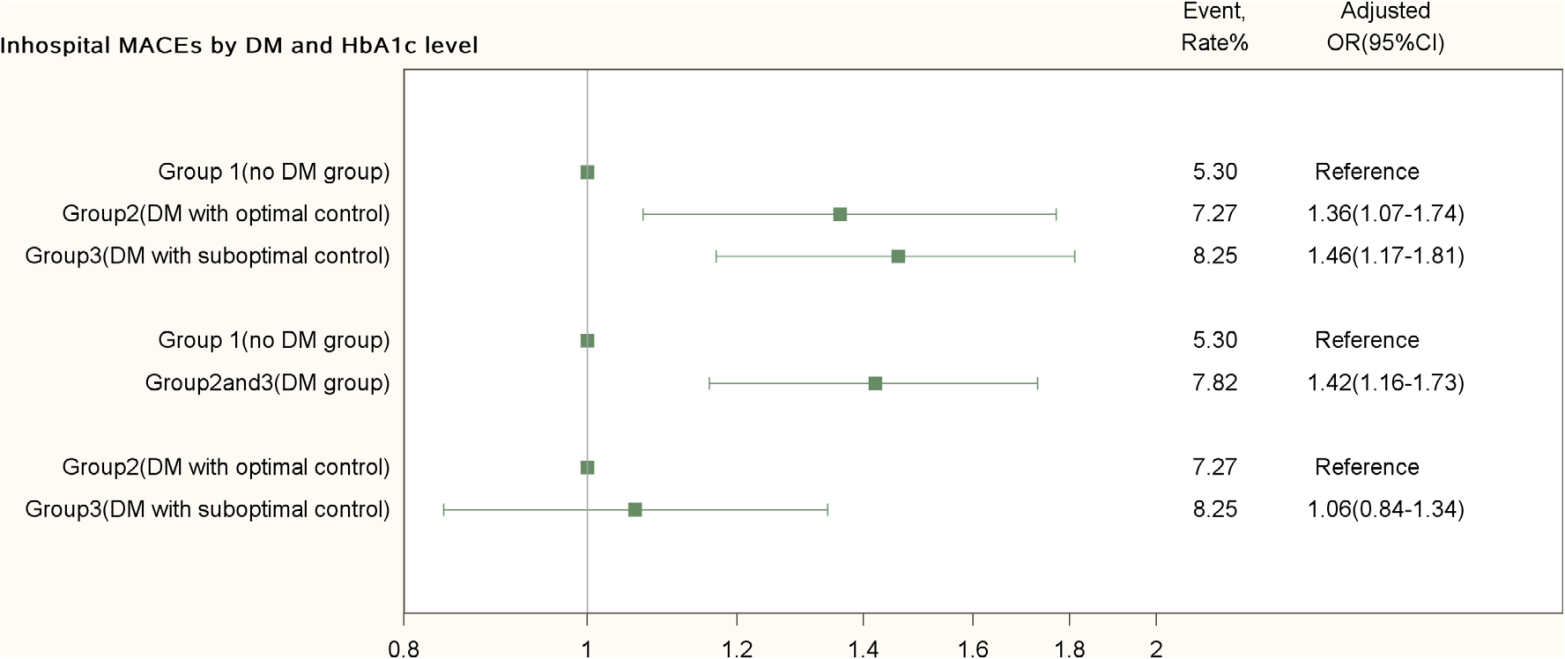
Associations of in‐hospital MACE with DM and HbA1c levels. ORs and their 95% CIs of DM and HbA1c levels for in‐hospital MACE were derived from multivariable logistic regression models, adjusting for gender, different subtypes of ACS, GRACE risk score, history of OMI, history of stroke/TIA, risk factors of cardiovascular disease, and in‐hospital treatment

Adjusted ORs (95% CI) stratified by diabetes and HbA1c levels of in‐hospital MACEs are provided (Figure 2). The primary end point (in‐hospital MACEs) occurred in 8.25% of patients in the DM with suboptimal control group (group 3) compared with 5.30% of patients in no DM group (group 1). The adjusted OR between group 3 and group 1 was 1.46 (1.17‐1.81), *P* = .001. There was also significant difference of in‐hospital MACEs between group 2 and group 1 (the adjusted OR 1.36[1.07‐1.74], *P* = .01). In addition, the primary end point occurred in 7.82% of patients in group 2 and group 3 compared with 5.30% of patients in group 1 (the adjusted OR 1.42[1.16‐1.73], *P* = .001). According to guidelines, group 2 and group 3 were DM patients with different HbA1c control conditions. Since we aimed to define the impact of different HbA1c levels (glycemic control) in DM on in‐hospital MACEs, additional adjusted OR between group 2 and group 3 was calculated (the adjusted OR 1.06[0.84‐1.34], *P* = .63).

### 4.3 Long‐term all‐cause and cardiac mortality stratified by diabetes and HbA1c level

**FIGURE 3:**
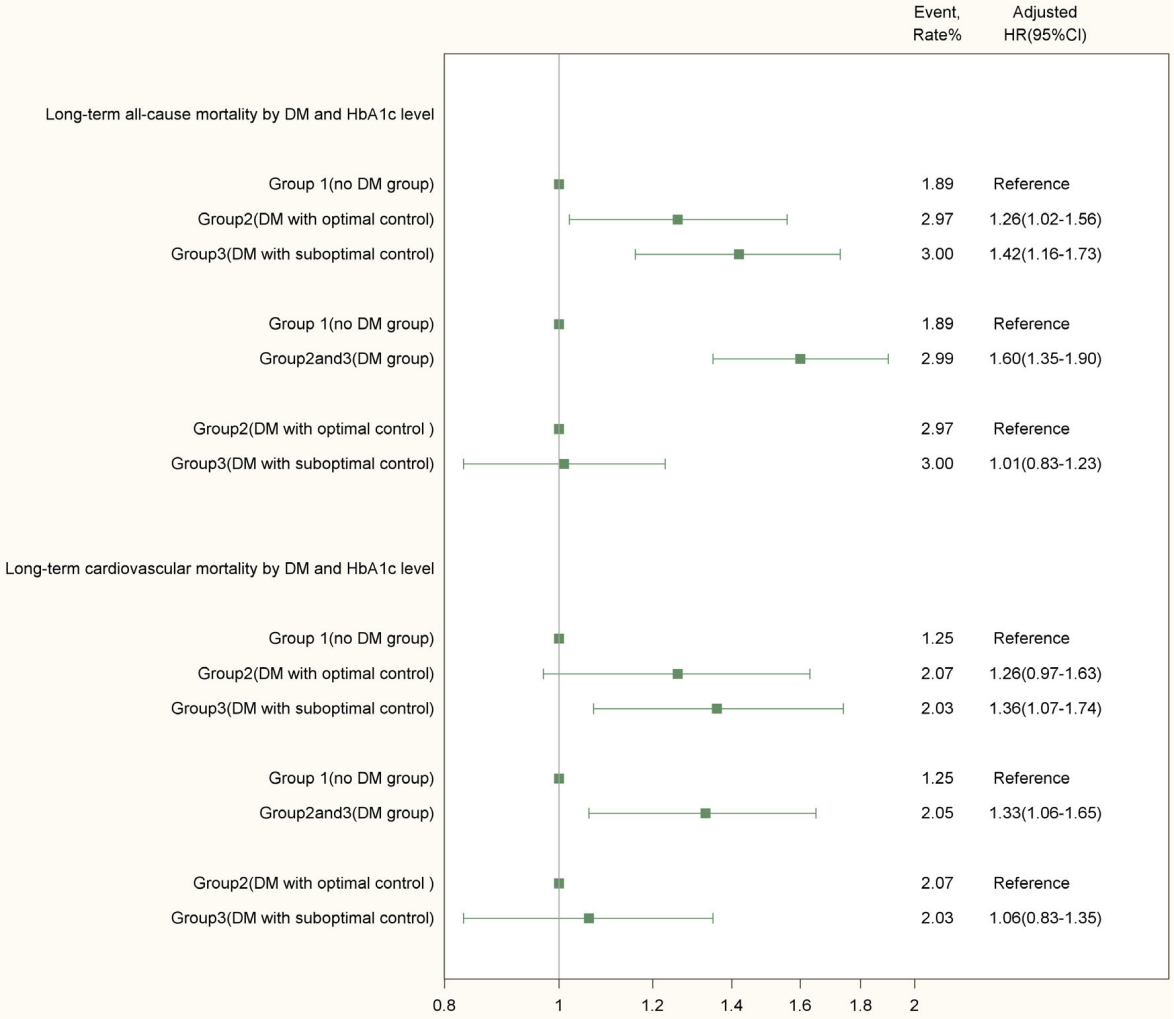
Associations of long‐term all‐cause/cardiac mortality with DM and HbA1c levels. HRs and their 95% CIs of long‐term all‐cause/cardiac mortality were derived from multivariable Cox proportional hazards models, adjusting for potential confounders that considered in the GRACE risk score or in CPACS risk score

The all‐cause mortality during a median follow‐up period of 3.85 years, was more common among group 2 (2.97% vs 1.89%; HR 1.26, 95% CI 1.02‐1.56) and group 3 (3.00% vs 1.89%; HR 1.42, 95% CI 1.16‐1.73) than among group 1 patients. This trend was also shown in long‐term cardiac mortality (Figure 3).

At the longest follow‐up period of 8 years, the adjusted cumulative all‐cause mortality was 3.00% in group 3 patients, 2.97% in group 2 patients, and 1.89% in the controls without diabetes (group 1). There was no significant difference between group 2 and group 3. The adjusted cumulative cardiac mortality at 8 years was 2.03% in group 3 patients, 2.07% in group 2 patients, and 1.25% in group 1 patients (Figure 3).

### 4.4 Propensity score matching analysis

We also performed an analysis with propensity score matching and showed the results in Table S1 and S2 of Supplement Material. Associations were consistent and showed the same trend with the main results.

### 4.5 Subgroup and sensitivity analysis

Associations were presented in subgroups defined according to the subtypes of ACS, that is, ST elevation myocardial infarction (STEMI) and non‐ST elevation acute coronary syndrome (NSTEACS) (Table S3 and S4). In brief, the subsets of STEMI in group 2 and group 3 were associated with greater in‐hospital MACEs, but the subgroups of NSTEACS showed the same trend in long‐term outcomes.

Thousand five hundred and twenty‐nine patients who had no previous history of DM, but had elevated HbA1c level ≥6.5% (newly diagnosed DM), were included in group 2 (873 patients) and group 3 (656 patients). We ran a sensitivity analysis by including only these patients and found the associations with in‐hospital MACEs were consistent in these patients (Table S5).

Furthermore, we also performed another sensitivity analysis by excluding all these patients (newly diagnosed DM) in group 2 and group 3, and found that associations were consistent in the remaining patients (Table S6).

## 5 DISCUSSION

This large observational study of 8961 Chinese patients with ACS showed that DM with both optimal and suboptimal glycemic control (different HbA1c levels) were associated with higher in‐hospital events and long‐term mortality than the ACS patients without DM. Moreover, glycemic control in DM with ACS (ie, controlling optimal HbA1c level <7.0%), which has been proved to be beneficial to lowering microvascular events, seemed to have limited impact on in‐hospital macrovascular events and long‐term mortality in the high‐risk population. As far as we know, this is the first study to determine the combined effect of overt DM and different HbA1c conditions (optimal or suboptimal control) on in‐hospital and long‐term clinical outcomes in Chinese patients with ACS.

### 5.1 Baseline characteristics

According to the diagnostic criteria of DM in the latest 2020 ADA recommendation (HbA1c ≥ 6.5%), our study could identify newly diagnosed DM patients.^13^ Among 5302 patients without history of DM, there were 1529 patients with HbA1c ≥6.5%. That's to say the definite DM (3659) and newly diagnosed DM (1529) occurred 57.9% in the whole elected ACS population (8961). This percentage of ACS patients with DM in our study (57.9%) was comparatively higher than the rate in another large multinational observational registry‐the GRACE (23.8%).^14^ However, it was nearly the same with the proportion in the China Heart Survey (59% DM in CVD patients).^15^ The possible reasons are as follow, firstly our study only included ACS patients with HbA1c levels which were just detected for patients with high risk of DM, and secondly DM was more prevalent in Chinese patients with ACS than in the Western ACS population.

### 5.2 The impact of combined DM and HbA1c level on in‐hospital or short‐term outcomes of patients with ACS

HbA1c levels during the index admission can reflect the average blood glucose levels and glycemic control during the previous 2 to 3 months before the hospitalization of ACS. In our study, whether or not there is optimal glucose control (HbA1c <7%), DM patients with ACS (group 2 and group 3) was associated with higher in‐hospital MACEs than ACS patients without DM (group 1). There were several studies which were designed to analyze the relationship of HbA1c level and short‐term outcomes of patients with ACS.^16^, ^17^, ^18^ Since the diagnostic criteria of DM with HbA1c levels had not been recommended at that time, these studies could not show the short‐term prognostic value of combine both DM and HbA1c levels in ACS patients. In addition, the samples of these studies were relatively small and could not reflect contemporary treatment. Giraldez et al. studied a large sample (8795 patients with NSTEACS) and found undiagnosed diabetes was associated with greater short‐term (30 days) death or myocardial infarction.^19^ We also found the significant association of DM with higher in‐hospital MACEs in Chinese patients with ACS, even after multivariable adjustment with potential confounders including baseline characteristics, GRACE risk score, history of cardiac disease, history of stroke/TIA, risk factors of cardiovascular disease and in‐hospital treatments. The mechanism of DM patients with higher short‐term events may be explained by insulin resistance, oxidative stress, as well as enhanced platelet activation.^20^, ^21^ One study also reported impaired coronary flow to be associated with high blood glucose in ACS patients.^22^

In our study, an additional investigation about association of glycemic control and in‐hospital events between DM with optimal control and DM with suboptimal control (group 2 vs group 3) showed insignificant result. This supported the Examination of Cardiovascular Outcomes with Alogliptin vs Standard of Care (EXAMINE) which included 5380 patients with type 2 diabetes and a recent ACS event.^23^ Heller et al. found no relationship between HbA1c levels (glycemic control) and short‐term MACEs (1 month) in DM patients with ACS.^23^ Optimal control defined DM patients with HbA1c <7%, they found no increase in risk of MACE with groups of higher HbA1c levels. It contrasts with a previous trial of saxagliptin vs placebo in comparably lower risk patients with type 2 diabetes mellitus (T2DM) and stable CVD or atherosclerotic risk factors (SAVOR‐TIMI 53), which suggested that groups with higher baseline HbA1c (>7%) were related with a higher risk of MACE.^24^ This may imply different risk populations: those in the EXAMINE study, who were 45 days from a previous ACS, had MACE at a proportion about double of that in populations in the SAVOR‐TIMI 53 trial, and the increased cardiac risk may have covered up a considerable impact of glycemic control.

### 5.3 The impact of combined DM and HbA1c level on long‐term outcomes of patients with ACS

We found that DM patients with ACS (both group 2 and group 3) were associated with higher long‐term mortality than ACS patients without DM (group 1) during a median follow‐up period of 3.85 years. It is in accordance with a recent large‐scale study. Stam‐Slob et al. found patients with T2DM and established CVD had a particularly high risk for MACE (the risk increased by about 1.7‐fold).^25^

Although the ACS patients with DM showed worse long‐term outcome, our study suggest that optimal glycemic control would not improve the long‐term mortality. It is in accordance with the Action to Control Cardiovascular Risk in Diabetes (ACCORD) trial, which planned to investigate that optimal or intensive glucose control could reduce MACEs in patients with T2DM and cardiac risk factors.^26^ However, the trial was terminated prematurely about 3.5 years because there were 22% more mortality in patients who were controlled intensively.^26^ Nowadays, several studies which were designed to reduce the MACEs in this high‐risk population with glucose lowering agents suggest only specific anti‐diabetic drugs decrease MACEs,^27^, ^28^, ^29^, ^30^ in addition the impact indicates not to be associated with HbA1c level.^27^, ^28^

A recent data point could partially explain the results. Savonitto et al. defined the predictors of all‐cause as well as cardiovascular mortality from large randomized clinical trial enrolling hospital survivors with T2DM and an ACS.^31^ The eight independent predictors of all‐cause mortality at a median follow‐up period of 2 years were defined with Cox regression analysis. The prediction by every variable was calculated with percentage of each chi‐square in the model. Markers of cardiac predictors included N‐terminal pro B‐type natriuretic peptide (27%), lack of coronary revascularization (18%), heart rate (10%), prior coronary artery bypass (7%) and prior myocardial infarction (6%), which totally showed 63% of prediction for all‐cause mortality. However, metabolic dysfunction just showed 8% of prediction. So mortality prediction of patients with T2DM and recent ACS is largely dominated by cardiovascular markers, rather than the metabolic marker (glycemic control or HbA1c level).

### 5.4 Different subtypes of ACS with DM and elevated HbA1c

Subgroup analysis showed that different types of ACS were associated with different timing of adverse events. The subsets of STEMI in group 2 and group 3 were associated with greater in‐hospital MACEs (short‐term outcomes), but the subgroups of NSTEACS showed the same significant trend in long‐term outcomes.

The temporal distribution of adverse events in patients with different subtype of ACS has been demonstrated in several studies.^32^, ^33^, ^34^

Typically, the risk of adverse events and mortality in patients affected by STEMI is the highest during the first month and then alleviates over time. This timing distribution is completely different with patients diagnosed as NSTEACS, who usually feature a higher risk of longer‐term outcome.^34^

## 6 LIMITATIONS

There are several limitations of our study. Firstly, the patients without HbA1c levels during the index admission were excluded. This may cause selection bias. Secondly, the duration of DM patients diagnosis, anti‐diabetic agents usage and HbA1c levels in the whole follow‐up duration cannot be defined. A few studies have showed the significance of long‐standing DM and cumulative hyperglycemic damages among patients on the danger for adverse cardiovascular events.^35^, ^36^ Although the DM patients with ACS in our study also showed the same trend during the long follow‐up period, the exactly timing effect of DM is worth further studies. Finally, we could not accurately define prediabetes in our population. These patients mostly were included in no DM group (group 1). However, in an analysis from the Providing Regional Observations to Study Predictors of Events in Coronary Tree (PROSPECT) study, authors defined prediabetes in population with ACS after successful PCI and assessed the related risk of MACEs. They concluded that DM but not prediabetes is associated with an increased risk for MACEs. Data demonstrated that DM was an independent predictor of MACEs, although patients with neither DM nor prediabetes had more severe CVD than no DM group.^37^

## 7 CONCLUSION

ACS with DM should be worth additional attention since these patients were associated with higher in‐hospital MACEs and long‐term mortality. Moreover, optimal glucose control and controlling HbA1c level, which has been proved to be beneficial to lower microvascular events, seems to have limited impact on macrovascular events and long‐term mortality in this specific high‐risk population. Thus, it is more important to get a therapeutic strategy for additional benefit of reducing cardiovascular events, not just anti‐diabetic treatment in DM patients with ACS.

## Supporting information


**APPENDIX** S1: Supporting Information
