# Supplementary material for: Impact of diabetes mellitus and hemoglobin A1c level on outcomes among Chinese patients with acute coronary syndrome
Source: Clin Cardiol. 2020 May 19;43(7):723–31. doi: 10.1002/clc.23373 (PMC7368303; doi:10.1002/clc.23373)
Supplement: Supplementary file 1 — APPENDIX S1: Supporting Information [file CLC-43-723-s001.docx]

**Supplemental material**

Table S1. Associations of in-hospital MACEs with DM and HbA1c levels using propensity score matched data (greedy nearest neighbor matching, caliper 0.1)

| In-hospital outcome | Group1  N=1689 | Group2  N=1689 | Group3  N=1689 | Adjusted OR(95%CI)  /P value | | | |
| --- | --- | --- | --- | --- | --- | --- | --- |
|  |  |  |  | Group2/Group1 | Group3/Group1 | Group2+Group3/Group1 | Group3/Group2 |
| In-hospital MACEs, n(%) | 84(4.97) | 107(6.34) | 107(6.34) | 1.27(0.94-1.70)  /0.12 | 1.32(0.98-1.78)  /0.06 | 1.29(1.00-1.68)  /0.05 | 1.04(0.79-1.38)  /0.77 |

Abbreviations: MACEs, major adverse cardiovascular events; OR, odds ratio.

Table S2. Associations of long-term all-cause/cardiac mortality with DM and HbA1c levels using propensity score matched data (greedy nearest neighbor matching, caliper 0.1)

| Long-term outcome | Group1  N=1198 | Group2  N=1198 | Group3  N=1198 | Adjusted HR(95%CI)  /P value | | | |
| --- | --- | --- | --- | --- | --- | --- | --- |
|  |  |  |  | Group2/Group1 | Group3/Group1 | Group2+Group3/Group1 | Group3/Group2 |
| All-cause mortality, n(%) | 102(2.32) | 134(2.95) | 140(3.08) | 1.29(0.99-1.66)  /0.06 | 1.34(1.04-1.73)  /0.03 | 1.31(1.05-1.65)  /0.02 | 1.04(0.82-1.32)  /0.75 |
| Cardiac mortality, n(%) | 71(1.61) | 93(2.05) | 85(1.87) | 1.28(0.94-1.75)  /0.12 | 1.17(0.85-1.60)  /0.34 | 1.22(0.93-1.61)  /0.15 | 0.91(0.68-1.22)  /0.53 |

Abbreviation: HR, hazard ratio.

Table S3. Subgroup analysis by different diagnose on in-hospital MACEs with DM and HbA1c levels using multivariable logistic regression models

| In-hospital outcome | Diagnose | Adjusted OR(95%CI)/P value | | | |
| --- | --- | --- | --- | --- | --- |
|  |  | Group2/Group1 | Group3/Group1 | Group2+Group3/Group1 | Group3/Group2 |
| In-hospital MACEs | STEMI | 1.51(1.13-2.03)  /p=0.005 | 1.47(1.14-1.91)  /p=0.004 | 1.49(1.18-1.88)  /p=0.001 | 0.98(0.73-1.30)  /p=0.87 |
|  | NSTEACS | 1.05(0.66-1.66)  /p=0.85 | 1.43(0.94-2.18)  /p=0.10 | 1.25(0.85-1.84)  /p=0.25 | 1.32(0.86-2.02)  /p=0.21 |

Abbreviations: MACEs, major adverse cardiovascular events; STEMI, ST elevation myocardial infarction; NSTEACS, non-ST elevation acute coronary syndrome; OR, odds ratio.

Table S4. Subgroup analysis by different diagnose on long-term all-cause/cardiac mortality with DM and HbA1c levels using multivariable COX proportional hazards models

| Long-term outcome | Diagnose | Adjusted HR(95%CI)/P value | | | |
| --- | --- | --- | --- | --- | --- |
|  |  | Group2/Group1 | Group3/Group1 | Group2+Group3/Group1 | Group3/Group2 |
| All-cause mortality | STEMI | 1.33(0.98-1.80)  /p=0.07 | 1.24(0.93-1.65)  /p=0.15 | 1.26(0.98-1.63)  /p=0.07 | 0.91(0.67-1.24)  /p=0.56 |
|  | NSTEACS | 1.27(0.94-1.71)  /p=0.12 | 1.65(1.24-2.21)  /p=0.001 | 1.43(1.11-1.86)  /p=0.007 | 1.29(0.99-1.70)  /p=0.06 |
| Cardiac mortality | STEMI | 1.28(0.87-1.86)  /p=0.21 | 1.20(0.85-1.70)  /p=0.31 | 1.22(0.89-1.67)  /p=0.21 | 0.90(0.62-1.31)  /p=0.59 |
|  | NSTEACS | 1.33(0.93-1.90)  /p=0.12 | 1.58(1.11-2.24)  /p=0.01 | 1.44(1.05-1.98)  /p=0.03 | 1.19(0.86-1.64)  /p=0.30 |

Abbreviations:STEMI, ST elevation myocardial infarction; NSTEACS, non-ST elevation acute coronary syndrome; HR, hazard ratio.

Table S5. Sensitivity analysis by including only the patients with elevated HbA1c and no previous history of DM in group2 and group3 on in-hospital MACEs using multivariable logistic regression models

| In-hospital outcome | Adjusted OR(95%CI)/P value | | | |
| --- | --- | --- | --- | --- |
|  | No previous history of DM in Group2/Group1 | No previous history of DM in Group3/Group1 | No previous history of DM in Group2 and Group3/Group1 | No previous history of DM in Group3/Group2 |
| In-hospital MACEs | 1.37(0.98-1.90)  /p=0.06 | 1.49(1.05-2.13)  /p=0.03 | 1.42(1.09-1.86)  /p=0.01 | 1.09(0.71-1.69)  /p=0.69 |

Abbreviations: MACEs, major adverse cardiovascular events; DM, diabetes mellitus; OR, odds ratio.

Table S6. Sensitivity analysis by excluding the newly detected patients in group2 and group3 on in-hospital MACEs using multivariable logistic regression models

| In-hospital outcome | Adjusted OR(95%CI)/P value | | | |
| --- | --- | --- | --- | --- |
|  | Previous history of DM in Group2/Group1 | Previous history of DM in Group3/Group1 | Previous history of DM in Group2 and Group3/Group1 | Previous history of DM in Group3/Group2 |
| In-hospital MACEs | 1.37(1.03-1.81)/p=0.03 | 1.46(1.16-1.85)/p=0.002 | 1.43(1.15-1.76)/p=0.001 | 1.07(0.81-1.34)/p=0.63 |

Abbreviations: MACEs, major adverse cardiovascular events; DM, diabetes mellitus; OR, odds ratio.
